# Enhanced *C. auris* screening and rapid diagnostics in cluster units: association with fewer incident clinical cases

**DOI:** 10.1017/ash.2026.10378

**Published:** 2026-05-19

**Authors:** Mayar Al Mohajer, Todd Lasco

**Affiliations:** 1 University of Oxford, Oxford, UK; 2 Baylor College of Medicine, USA

## Abstract

*Candida auris* screening in units meeting a prespecified cluster threshold was intensified from biweekly to weekly and paired with rapid in-house PCR (November 2022–March 2024). Monthly incident clinical cases decreased by 32%. Colonization detections increased with weekly screening and declined after in-house testing, supporting earlier case finding and response.

## Background

The emerging yeast *Candida auris* (hereafter *C. auris*) is notable for multidrug resistance and its propensity for prolonged healthcare-associated transmission.^1^ A recent taxonomic revision reclassified *C. auris* as *Candidozyma auris*; however, most clinical and public health guidance continues to use the historical nomenclature.^2^

In acute-care hospitals and post-acute facilities, *C. auris* colonizes the skin (particularly the axilla and groin) and can persist for months, often without symptoms.^3^ Because colonization is associated with environmental contamination and patient-to-patient spread, early identification of colonized patients is central to containment during unit-level cluster response.^3^

The Centers for Disease Control and Prevention (CDC) recommends targeted screening of high-risk admissions and contacts of newly identified cases, with serial point-prevalence surveys when there is evidence of ongoing transmission.^4^ We evaluated whether intensifying screening from biweekly to weekly in cluster units and implementing in-house PCR were associated with changes in monthly incident clinical *C. auris* cases.

## Methods

We conducted a retrospective quality-improvement evaluation at a quaternary academic medical center in Texas from November 2022 through March 2024. All inpatient rooms were single occupancy. Medical-surgical and intensive care units were designated as cluster units when ≥3 healthcare-associated *C. auris* cases occurred within a 4-week period. Healthcare-associated cases were defined as the first positive screening or clinical specimen obtained >24 hours after admission in a patient with no prior documented history of *C. auris*.

Patients were screened if they were (1) housed in a cluster unit (point-prevalence surveys), (2) transferred from a long-term acute-care hospital, or skilled nursing facility in the prior year, (3) epidemiologically exposed (overlap with a healthcare-associated case or missed isolation for a known positive transfer), or (4) on select high-risk services (eg, periodic transplant/oncology screening). A separate screen based solely on recent overnight stays in international healthcare facilities was not part of the institutional protocol. Cluster-unit surveys occurred on Mondays. Nursing staff identified eligible patients, and a single composite swab of the axilla and groin was collected for each screening test.^4^ Weekend high-risk admissions were generally screened on Monday; short-stay discharges before Monday screening were not captured separately.

Screening phases were biweekly (November 2022–February 2023), weekly (March–December 2023), and weekly with in-house PCR (January–March 2024). Send-out PCR results were available to clinicians in ∼4–5 days; in-house PCR reported within hours, but with a capacity of ∼30–40 tests/day, large cluster surveys required 2–4 days to complete. High-risk admissions were placed in Contact Precautions pending screening results. Within cluster units, patients without known *C. auris* positivity were managed with enhanced precautions, defined as empiric escalation of barrier precautions and environmental disinfection while screening was in progress; Contact Precautions were used for confirmed cases and for patients leaving the cluster unit until a negative screen was documented. Rooms of patients on Contact Precautions received daily and terminal cleaning with sodium hypochlorite disinfectant, and shared equipment was disinfected between uses with quaternary ammonium compound–isopropyl alcohol wipes.

The primary outcome was the monthly number of incident clinical *C. auris* cases, defined as the first non-surveillance clinical isolate per patient; urine and sputum isolates were excluded. Secondary outcomes were monthly colonization detections identified by screening PCR and by urine or sputum cultures. Colonization detections were interpreted as an operational yield measure because the first survey after cluster designation may also identify prevalent colonization. Monthly outcomes were monitored using statistical process control c-charts with phase-specific centerlines, and effects were summarized as percentage change compared with baseline. This study was approved by the institutional review board at Baylor College of Medicine.

## Results

Thirty-eight incident clinical cases were identified during the study period. Median patient age was 55 years (interquartile range [IQR], 44–65), and 66% were male. Clinical isolates were most commonly from blood (18/38, 47%), wound or tissue (17/38, 45%), and other body fluids (3/38, 8%). Fifteen clinical cases (39%) occurred in patients with a prior positive surveillance test, with a median of 27 days (IQR, 17–120) from first positive screen to clinical isolate. Screening and precaution adherence remained >90% during the study period based on nursing audits with infection prevention verification.

**Figure 1.**
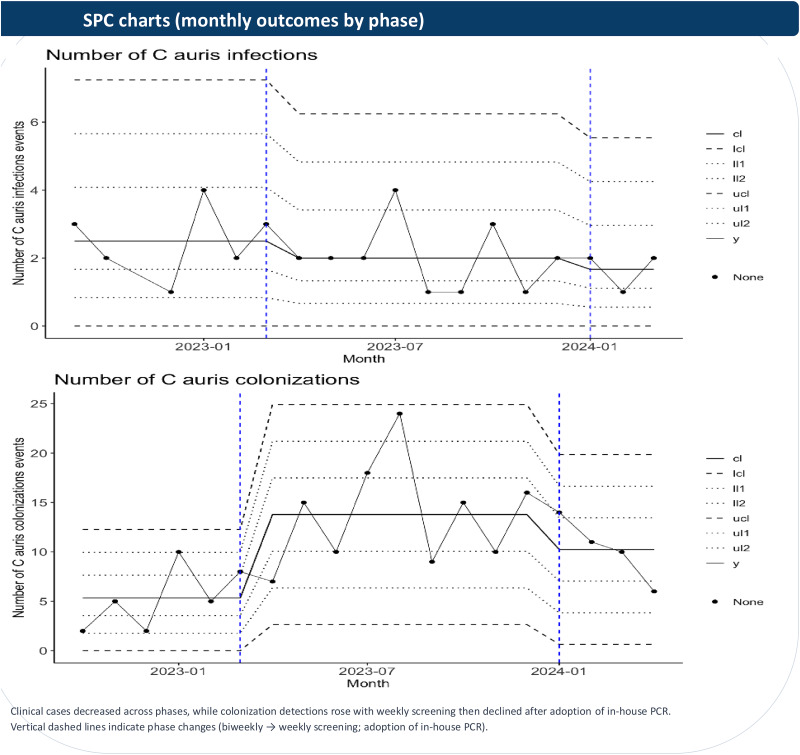
Monthly clinical cases and colonization detections by screening/diagnostic phase. Figure displayed as statistical process control c-charts for quality-improvement monitoring. Vertical dashed lines indicate phase changes (biweekly to weekly screening; adoption of in-house PCR).

As shown in Table 1, mean monthly incident clinical cases decreased from 2.5 cases/month during biweekly screening (phase 1) to 2.0 cases/month with weekly screening (phase 2; −20% vs baseline) and to 1.7 cases/month after adoption of in-house PCR (phase 3; −32% vs baseline). Mean monthly colonization detections increased from 5.3/month in phase 1 to 13.8/month in phase 2 (+160%) and then declined to 10.2/month after adoption of in-house PCR (+92% vs baseline; −26% vs phase 2). Temporal trends are shown in Figure 1.

**Table 1.** Mean monthly outcomes by screening/diagnostic phase

Phase	Strategy	Mean monthly clinical casesa	Mean monthly colonization detectionsb
1 (baseline)	Targeted screening every 2 weeks	2.5 (reference)	5.3 (reference)
2	Targeted weekly screening	2.0 (−20%)	13.8 (+160%)
3	Weekly screening + in-house PCR	1.7 (−32%)	10.2 (+92% vs baseline; −26% vs phase 2)

PCR, polymerase chain reaction.

^a^Clinical cases were defined as new non-surveillance clinical isolates; urine and sputum isolates were excluded.

^b^Colonization detections were identified by surveillance PCR and by urine/sputum cultures.

## Discussion

In cluster units meeting a threshold of ≥3 healthcare-associated *C. auris* cases within 4 weeks, intensifying surveillance from biweekly to weekly and transitioning to in-house PCR were associated with stepwise decreases in monthly incident clinical cases. Weekly screening increased colonization detections, consistent with improved case finding, whereas faster in-house testing was followed by fewer clinical cases and fewer colonization detections despite unchanged screening frequency.

We intentionally used incident clinical cases rather than proven infections as the primary outcome because some non-urine/non-sputum clinical isolates may represent colonization in the context of clinical sampling. The increase in colonization detections with weekly screening likely reflects improved case finding, especially because the first survey after cluster designation may establish the baseline burden already present on the unit. The subsequent decline after adoption of in-house testing may indicate earlier containment, but differences in test performance between the send-out and in-house PCR assays may also have contributed to lower colonization yield.

Published experience indicates that moving from send-out to on-site PCR can shorten time to colonization detection from days to hours and may reduce isolation days or costs and, in some settings, hospital-onset fungemia rates.^5–7^ In our hospital, clinical reporting improved from ∼4–5 days to 2–3 days for full cluster surveys because throughput (30–40 tests/day) limited completion; this underscores the importance of end-to-end turnaround time, including weekends and batching, not just analytical run time.

Nearly 40% of incident clinical cases were preceded by a positive screen with a median interval of 27 days, suggesting a window to sustain precautions and optimize device, wound, and transfer management; however, phase-specific estimates of preclinical detection were unavailable.^8–10^ This single-center phased implementation study cannot establish causality. Outcomes were monthly counts rather than patient-day–adjusted rates, and patient-days, admissions, numbers screened, test positivity, time to isolation, contact-precaution prevalence, unit characteristics, and concurrent infection prevention activities were unavailable. We did not perform culture confirmation or molecular typing, and findings may not generalize to centers with different patient populations, baseline prevalence, or laboratory capacity.

In settings experiencing *C. auris* clusters, weekly targeted screening paired with faster operational PCR turnaround time may enable earlier identification and response. Future work should incorporate denominator data, phase-specific progression from colonization to clinical case, and end-to-end turnaround time including weekend coverage.

## Data Availability

Deidentified aggregate data supporting the findings are available from the corresponding author upon reasonable request and consistent with institutional policies.
